# Data on draft genome sequence of *Caldanaerobacter* sp. strain 1523vc, a thermophilic bacterium, isolated from a hot spring of Uzon Caldera, (Kamchatka, Russia)

**DOI:** 10.1016/j.dib.2020.106336

**Published:** 2020-09-24

**Authors:** A.A. Korzhenkov, S.V. Toshchakov, O.A. Podosokorskaya, M.V. Patrushev, I.V. Kublanov

**Affiliations:** a National Research Center "Kurchatov Institute", Moscow 123182, Russia; b Winogradsky Institute of Microbiology of Federal Research Centre “Fundamentals of Biotechnology” of the Russian Academy of Sciences, Russia, 117312, Moscow, 60-let Oktyabrya prospect 7/2

**Keywords:** Thermophiles, CAZymes, *Caldanaerobacter*, Genome, Extremophiles

## Abstract

The draft genome sequence of *Caldanaerobacter* sp. strain 1523vc, a thermophilic bacterium, isolated from a hot spring of Uzon Caldera, (Kamchatka, Russia) is presented. The complete genome assembly was of 2 713 207 bp with predicted completeness of 99.38%. Genome structural annotation revealed 2674 protein-coding genes, 127 pseudogenes and 77 RNA genes. Pangenome analysis of 7 currently available high quality *Caldanaerobacter* spp. genomes including 1523vc revealed 4673 gene clusters. Of them, 1130 clusters formed a core genome of genus *Caldanaerobacter*. Of the rest 3543 *Caldanaerobacter* pangenome genes, 385 were exclusively represented in 1523vc genome. 101 of 2801 *Caldanaerobacter* CDS were found to be encoding carbohydrate-active enzymes (CAZymes). The majority of CAZymes were predicted to be involved in degradation of beta-linked polysaccharides as chitin, cellulose and hemicelluloses, reflecting the metabolism of strain 1523vc, isolated on cellulose. 5 of 101 CAZyme genes were found to be unique for the strain 1523vc and belonged to GH23, GT56, GH15 and two CE9 family proteins.

The draft genome of strain 1523vc was deposited at DBJ/EMBL/GenBank under the accessions JABEQB000000000, PRJNA629090 and SAMN14766777 for Genome, Bioproject and Biosample, respectively.

## Specifications Table

**Table:** 

Subject	Biology, Microbiology
Specific subject area	Microbial biotechnology
Type of data	Genomic sequence, predicted genes and functional analysis of respective proteins
How data was acquired	*De novo* whole genome sequencing Instrument: Illumina MiSeq
Data format	Raw data: annotated draft genome assembly; Secondary data: table of orthologous gene clusters of *Caldanaerobacter* representatives; table of average nucleotide indentity between *Caldanaerobacter* genomes
Parameters for data collection	Thermophilic anaerobic pure culture cultivation. Extraction of genomic DNA from a pure culture, fragment library preparation, Illumina sequencing, *de novo* assembly and annotation procedures
Description of data collection	Extraction of genomic DNA was performed with ISOLATE II Genome DNA kit (Bioline, UK); fragment library was prepared with NEBNext Ultra kit; sequencing was performed with Illumina MiSeq™ system. The genome was assembled using Unicycler and annotated with NCBI PGAP web server
Data source location	The culture of strain 1523vc is deposited in extremophiles metabolism laboratory collection at Federal Research Center “Biotechnology” RAS (Moscow, Russian Federation)
Data accessibility	Raw data is publicly available at NCBI Genbank.The Biosample, Bioproject and assembly/WGS accession numbers are: SAMN14766777 (https://www.ncbi.nlm.nih.gov/biosample/SAMN14766777) PRJNA629090 (https://www.ncbi.nlm.nih.gov/bioproject/PRJNA629090) https://www.ncbi.nlm.nih.gov/bioproject/PRJNA511806) and JABEQB000000000 RZHM00000000 (https://www.ncbi.nlm.nih.gov/nuccore/JABEQB000000000), https://www.ncbi.nlm.nih.gov/nuccore/RZHM00000000), respectively. Secondary data is available as Supplementary Table 1 and 2.
Related research article	Kublanov IV, Perevalova AA, Slobodkina GB, et al. Biodiversity of thermophilic prokaryotes with hydrolytic activities in hot springs of Uzon Caldera, Kamchatka (Russia). *Appl Environ Microbiol*. 2009;75(1):286‐291. doi:10.1128/AEM.00607-08

## Value of the Data

• Genome data for Caldanaerobacter sp. 1523vc can be used for genome-based phylogenetic and evolutionary analysis of Caldanaerobacter genus
• 385 of 3543 Caldanaerobacter pangenome genes were found to be represented exclusively in strain 1523vc genome. Among them are several carbohydrate-active enzymes (CAZymes, http://www.cazy.org) attributed to GH23, GT56 and GH15 and two CE9 family proteins, which can be further explored by biotechnologists using heterologous expression and activity analysis
• The genome encodes a high number of CAZymes, participating in degradation of various beta-glucans, which could be relevant to various applications, including 2nd generation bioethanol production, as well as pulp and food industries. Genomic data, presented in this article unlock the coding potential of strain 1523vc for further biochemical analysis of its enzymes in the scope of biotechnological applications

## 1 Data Description

*Caldanaerobacter* is a genus of *Firmicutes* phylum, which was proposed by Fardeau et al., in 2004 upon isolation of two thermophilic bacterial strains and reclassification of three species, formerly representing the genus *Thermoanaerobacter* as well as *Carboxydibrachium pacificum* [1]. Later, a second species of the genus was proposed by Kozina and co-authors in 2010 [2]. The members of the genus are Gram-positive thermophilic strictly anaerobic chemoorganoheterotrophic bacteria, growing on carbohydrates and proteinaceous substrates. Among the biopolymers, known to be hydrolyzed by the genus members are xylan, starch and agarose [1], [2] as well as keratins [3], [4].

Strain 1523vc was isolated from an *in situ* enrichment culture proliferating on a linen rope in a 70°C hot spring, and it is a first *Caldanaerobacter* representative, capable of growing on microcrystalline and carboxymethyl cellulose [4].

**Fig. 1:**
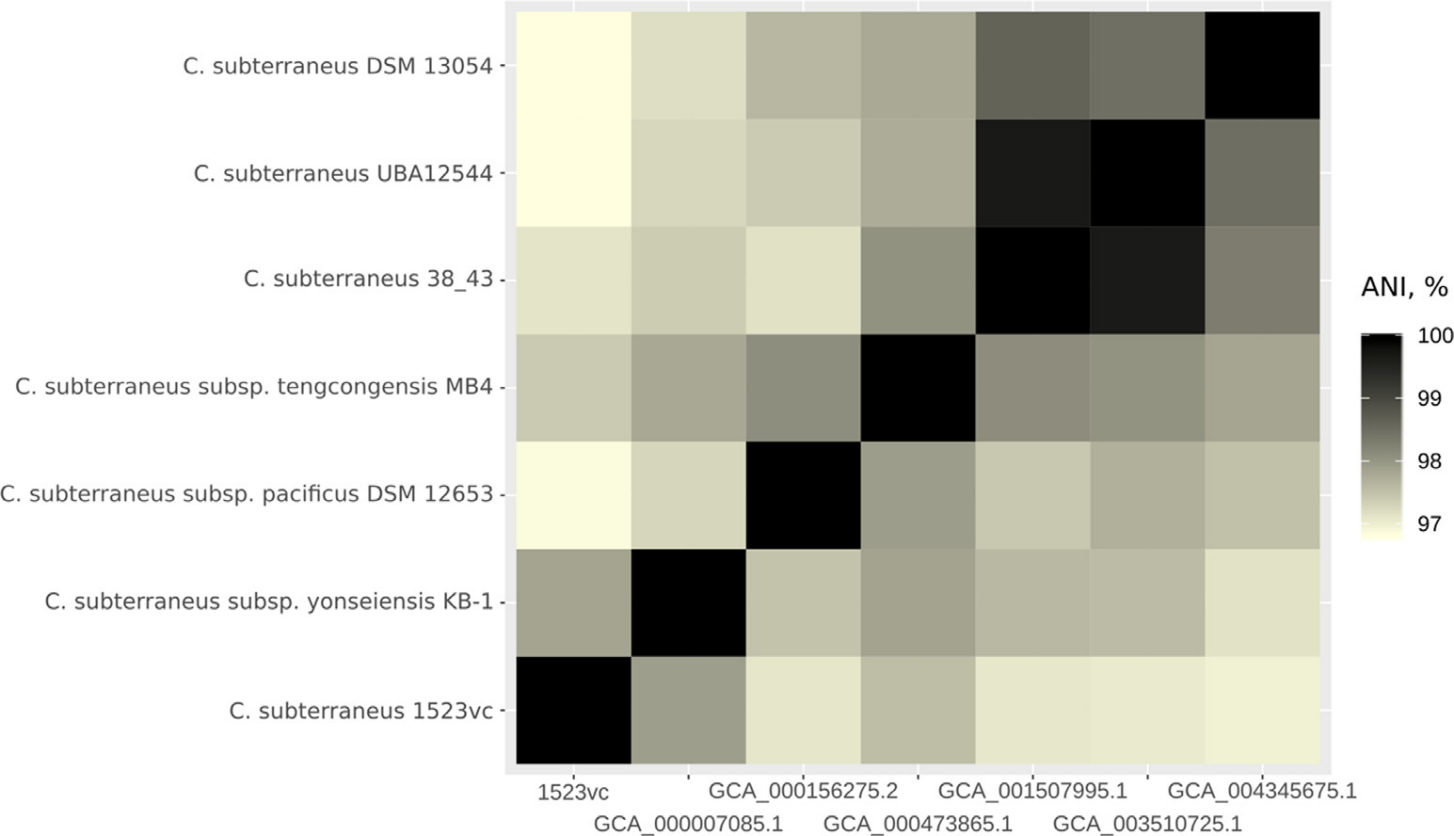
Heatmap of average nucleotide identity values between Caldanaerobacter strains, calculated by ani.rb script of enveomics package [10]. Strain IDs are shown at vertical axis, corresponding NCBI Assembly IDs are shown on horizontal axis. ANI data, used for this heatmap are presented in Supplementary Table 2.

Strain 1523vc genome was sequenced using Illumina MiSeq™ platform. The complete genome assembly was of 2 713 207 bp with GC-content of 37.2 mol%. Completeness of the assembly was estimated to be 99.38%. Analysis of average nucleotide identity of 1523vc and genomes of *Caldanaerobacter* spp. (Fig. 1, Supplementary Table 2) showed that strain 1523vc is closely related to *C.subterraneus* subsp. *yonseiensis,* which was also isolated from a geothermal hot spring [1], [5].

Genome annotation with NCBI Prokaryotic Genome Annotation Pipeline [6] revealed 2801 protein-coding sequences including 2674 CDSs, 127 pseudogenes, and 77 RNAs genes. Public genomic databases (NCBI, IMG) contain six high-quality *Caldanaerobacter* genome assemblies. Pangenome analysis of the seven *Caldanaerobacter* spp. genomes (including 1523vc) using ProteinOrtho [7] revealed 4673 gene clusters (Supplementary Table 1). Of them, 1130 clusters formed a *Caldanaerobacter* core genome. Of the rest 3543 *Caldanaerobacter* pangenome genes, 385 were exclusively represented in 1523vc genome. 92 of these genes were located in laterally acquired gene islands, detected by IslandViewer 4 [8].

101 of 2801 *Caldanaerobacter* CDS were found to be encoding CAZymes – the proteins, that degrade, modify, or create glycosidic bonds [9]. The most numerous families were CE9, CE14, CBM50, GH109, GT4. The majority of CAZymes were involved in degradation of beta-linked polysaccharides as chitin, cellulose and hemicelluloses reflecting the metabolism of strain 1523vc, isolated and growing on various cellulose substrates [4]. Of 101 CAZyme-related genes 5 glycozide hydrolazes and carbohydrate esterases were found to be unique for the strain 1523vc: HKI81_01480 and HKI81_01510 (CE9, adenine deaminase), HKI81_04210 (GH23, transglycosylase SLT domain-containing protein), HKI81_12285 (GT56, 4-alpha-L-fucosyltransferase) and HKI81_13925 (GH4, alpha-gluco/galactosidase). Thus, relatively small number of CAZymes, specific for strain 1523vc suggests consistent set of CAZymes within the *Caldananaerobacter* genus and hence, comparable capabilities to degrade polysaccharides within the genus members. Indeed, 30 CAZymes were found to be encoded by the *Caldanaerobacter* core genome genes, among which there were families with known cellulase (GH5), amylase (GH13), chitinase (GH18), lysozyme (GH23) and mannan phosphorylase (GH94) activities.

The draft genome of strain 1523vc was deposited at DBJ/EMBL/GenBank under the accessions JABEQB000000000, PRJNA629090 and SAMN14766777 for Genome, Bioproject and Biosample, respectively.

## 2 Experimental Design, Materials, and Methods

### 2.1. Strain isolation and deposition into collection

Strain 1523vc isolation procedure was described previously [4]. The strain is maintained in the extremophiles metabolism laboratory (Winogradsky Institute of Microbiology, now a part of FRC “Biotechnology”, RAS) collection by annual transfer on the medium, described previously [4]. For genomic sequencing one liter of the same medium was prepared, and strain 1523vc was cultivated in its optimal growth conditions. The grown cells were harvested by centrifugation at 12000 *g*.

### 2.1. DNA extraction, library preparation and sequencing

Genomic DNA was isolated using ISOLATE II Genome DNA kit (Bioline, UK). Fragmentation of genomic DNA was performed with Bioruptor™ sonicator (Diagenode, Belgium) to achieve an average fragment length of 400 bp. Further steps of library preparation were performed with NEBNext® Ultra™ fragment library kit (New England BioLabs) according to the manufacturer's instructions. Bead-based size-selection was performed to get fragment sizes in the range of 300–500 bp. Sequencing was done with Illumina MiSeq™ platform (Illumina, USA) using 300 cycles paired-end sequencing reagents. 1,600,832 read pairs were obtained from the sequencing run.

### 2.2. De novo assembly

Raw sequencing reads were trimmed by quality with CLC Genomics Workbench v. 10.0.1 (Qiagen, Germany). Adapter sequences were trimmed with SeqPrep tool (https://github.com/jstjohn/SeqPrep). Finally, 1,462,277 read pairs were used for the assembly. Genome was assembled with Unicycler v.0.4.8 [11]. Genome completeness and contamination were assessed with CheckM [12] using *Thermoanaerobacteraceae*-specific marker set.

### 2.3. Genome annotation and analysis

Genome was annotated with NCBI PGAP [6].Average nucleotide identity (ANI) was calculated using ani.rb script (https://github.com/lmrodriguezr/enveomics) [10]. ANI heatmap was plotted using ggplot2 library for R [13].CAZymes [9] were searched using hmmscan [14] in dbCAN v. 2.0 [15] followed by manual verification using hmmscan and Pfam databases [16].

## Declaration of Competing Interest

The authors declare that they have no known competing financial interests or personal relationships which have, or could be perceived to have, influenced the work reported in this article.

## Appendix Supplementary materials

Image, application 1

Image, application 2
